# Exploring the Well-Being, Adaptability, and Sense of Belonging of Undergraduate Nursing Students During the Transition From Simulation to Clinical Practice: Protocol for a Scoping Review

**DOI:** 10.2196/86813

**Published:** 2026-02-13

**Authors:** Marjolaine Dionne Merlin, Monica McGraw, Julie Renaud, Sandrine Evelyne Roy

**Affiliations:** 1 School of Nursing, Faculté des sciences de la santé et des services communautaires, Université de Moncton, 51 Avenue Antonine-Maillet, Moncton, NB, E1A 3E9, Canada, 1 5068584000 ext 2172

**Keywords:** nursing students, simulation-based learning, clinical education, well-being, adaptability, sense of belonging, transition, scoping review

## Abstract

**Background:**

The transition from university-based simulation learning to the clinical environment is a pivotal stage in undergraduate nursing education. This period can influence students’ psychological well-being, adaptability, and sense of belonging within the clinical setting, which are essential dimensions to professional learning and patient safety. Although simulations aim to prepare students for clinical realities, the extent to which they support students’ emotional and social readiness for real practice remains unclear.

**Objective:**

This scoping review aims to map literature on undergraduate nursing students’ well-being, adaptability, and sense of belonging during their transition from university-based simulation learning to clinical practice. Secondary objectives include identifying educational interventions and highlighting research gaps for future research.

**Methods:**

The review will follow the JBI methodology for scoping reviews and the PRISMA-ScR (Preferred Reporting Items for Systematic Reviews and Meta-Analyses extension for Scoping Reviews) reporting guidelines. A systematic search will be conducted in PubMed, CINAHL, APA PsycINFO, Scopus, and ScienceDirect for studies published between 2015 and 2025 in English or French. A gray literature search will also be performed in Google Scholar. Eligible studies will include empirical (quantitative, qualitative, or mixed method) and review papers exploring nursing students’ experiences of transitioning from university-based simulation learning to real clinical settings. Two reviewers will independently screen titles, abstracts, and full texts; extract relevant data; and synthesize findings using thematic analysis.

**Results:**

As of February 2026, data collection is projected to be completed by April 2026, and data analysis is expected to be finalized by June 2026. Results will summarize current definitions, measures, and interventions related to students’ well-being, adaptability, and sense of belonging, as well as identify evidence gaps and conceptual trends in literature.

**Conclusions:**

This scoping review will address a critical gap by clarifying how psychosocial dimensions of the transition from university-based simulation learning to clinical practice are conceptualized, measured, and supported in undergraduate nursing education, thereby informing the development of more comprehensive and learner-centered educational strategies.

## Introduction

The transition from university-based simulation learning to clinical practice marks a pivotal stage in undergraduate nursing education. Simulation has become a cornerstone of modern pedagogy, providing students with a safe and controlled environment to develop technical skills, clinical reasoning, and decision-making without compromising patient safety [1,2]. Through simulations, standardized patients, and immersive scenarios, educators strive to replicate the complexity of real-world care, equipping nursing students for the challenges of clinical experience. Yet, despite these advantages, moving from simulated settings to authentic clinical environments introduces challenges that go far beyond technical competence. This shift often demands significant psychological, social, and emotional adjustments, which can profoundly shape students’ learning experiences and professional growth [2].

Clinical placements expose students to unpredictable situations and interpersonal dynamics that differ markedly from the controlled conditions of university-based simulation learning. These changes can cause stress, anxiety, and feelings of uncertainty, particularly when students identify a gap between their simulated experiences and real-world expectations [3]. Research suggests that psychological well-being, adaptability, and sense of belonging are pivotal for successful integration into clinical settings, as these dimensions affect confidence, resilience, and ultimately patient safety [3,4]. Students who feel supported and included within the clinical team are more likely to engage actively in learning, demonstrate adaptability, and develop professional identity [5,6]. In contrast, lack of belonging or inadequate coping strategies can lead to disengagement, burnout, and attrition from nursing programs [6,7].

In this review, psychological well-being, adaptability, and sense of belonging are treated as distinct yet interrelated constructs. Psychological well-being is conceptualized as a multidimensional indicator of positive mental health encompassing emotional, psychological, and social dimensions, including life satisfaction, positive affect, and psychological functioning [8,9]. Although resilience is frequently examined alongside well-being in nursing student research, it is considered here as a related protective factor rather than a proxy for well-being. Adaptability is defined as the capacity to adjust cognitive, emotional, and behavioral responses to novel, uncertain, or changing academic and clinical demands [10]. In line with Waldeck et al [11], adaptability is distinguished from resilience in that it reflects ongoing adjustment processes rather than recovery following adversity. Sense of belonging is understood as a relational and context-dependent construct referring to individuals’ perceptions of being accepted, valued, and included within academic, clinical, or institutional environments [3,6,12]. While a sense of belonging is recognized as an important determinant of both well-being and academic persistence, it remains analytically distinct from individual adaptive capacities and emotional regulation processes [13].

Although these constructs are sometimes used interchangeably across studies, this review treats them as analytically distinct but potentially overlapping concepts that constitute a psychosocial foundation supporting nursing students’ competence development and professional socialization during the transition period.

Despite the recognized importance of these dimensions, evidence on how simulation prepares students for the psychosocial experiences of clinical practice remains insufficient. While simulation can enhance confidence and technical skills [14], its impact on emotional readiness and social integration is less clear. Some studies indicate that structured debriefing and reflective practice within simulation may promote resilience and coping strategies [14], yet others highlight persistent challenges when students encounter authentic clinical pressures [15]. Educational interventions such as orientation programs, mentorship, and peer support have been proposed to bridge this gap, but their effectiveness varies across contexts and remains underexplored in the literature.

Given these complexities, a comprehensive synthesis of existing research is needed to map what is known about nursing students’ well-being, adaptability, and sense of belonging during the transition from university-based simulation learning to clinical practice. Scoping reviews are particularly suited for this purpose, as they allow for the examination of diverse study designs, identification of conceptual frameworks, and analysis of interventions aimed at supporting students during this critical phase [16,17]. This study aims to systematically review empirical and theoretical contributions to clarify how key psychosocial dimensions—well-being, adaptability, and sense of belonging—are defined, measured, and supported during the transition from university-based simulation learning to clinical practice. By mapping existing evidence, the review will identify educational strategies that influence these outcomes, highlight gaps in the literature, and propose directions for future research to inform curriculum design and policy development in nursing education. Specifically, the primary objective is to synthesize research on nursing students’ experiences of this transition, while secondary objectives include examining interventions that foster psychosocial readiness and outlining areas requiring further investigation. To guide this work, the review will address three questions: (1) What is currently known about well-being, adaptability, and sense of belonging among undergraduate nursing students during the transition from university-based simulation learning to clinical practice? (2) Which interventions or contextual factors support these dimensions during this period? (3) What gaps persist in the literature, and what priorities should shape future research?

In summary, the transition from university-based simulation learning to clinical practice is a multidimensional process that shapes students’ professional identity and capacity for safe, effective care. Understanding how well-being, adaptability, and sense of belonging interact during this period is essential for designing interventions that promote successful integration into clinical environments. This scoping review will provide an evidence-informed foundation for enhancing educational practices and supporting nursing students in navigating one of the most challenging stages of their academic journey.

## Methods

### Overview

This scoping review will follow the JBI methodological framework for scoping reviews and will be reported in alignment with the PRISMA-ScR (Preferred Reporting Items for Systematic Reviews and Meta-Analyses extension for Scoping Reviews) checklist (Checklist 1). The review process will include the following stages: (1) defining the research question, (2) identifying relevant studies, (3) selecting eligible evidence, (4) charting the data, and (5) collating, summarizing, and reporting the results.

A comprehensive literature search will be conducted across major bibliographic databases and relevant gray literature sources (eg, dissertations, reports, association websites) to capture studies published between 2015 and 2025 in English and French. The search will be designed to retrieve studies addressing concepts related to nursing students, university-based simulation learning, clinical practice, well-being, adaptability, and sense of belonging. Reference lists of included publications will also be reviewed to identify additional evidence. Any significant updates to the protocol will be documented and presented in the final scoping review report. An initial exploratory search across relevant databases confirmed that no systematic or scoping reviews currently exist on this specific topic, nor are any in progress.

For this review, the “transition from simulation to clinical practice” is defined as the process through which learners move from university-based simulation learning to real-world clinical practice settings. This transition is not limited to a single point in time or to the first clinical placement but occurs at multiple moments throughout the undergraduate nursing program, as learners progressively engage in clinical experiences following simulation-based learning activities.

### Eligibility Criteria

Eligible studies include peer-reviewed empirical research (quantitative, qualitative, or mixed method) and review articles published in English or French between 2015 and 2025, reflecting the rapid evolution of university-based simulation learning in nursing over the past decade. Foundational studies published prior to 2015 informed the background of the review but will not be included in the formal synthesis. Review articles will be used primarily to map and clarify concepts, rather than to contribute quantitative or duplicated findings from primary studies. Data from reviews will support the identification of key constructs, definitions, and conceptual frameworks, while primary studies will remain the main source of empirical data for synthesis.

The papers must focus on undergraduate or prelicensure nursing students enrolled in nursing education programs and engaged in experiential learning activities such as clinical placements, simulation-based learning, or internships. Studies should explore the transition from university-based simulation learning to real clinical settings and may examine interventions such as orientation programs, mentoring, or structured debriefing that support psychological well-being, resilience, adaptability, sense of belonging, and integration into clinical practice. Comparisons across different learning environments (eg, simulation vs real-world practices) are acceptable, as are outcomes related to confidence, satisfaction, perceived competence, and other indicators of clinical learning.

### Search Strategy

An academic librarian at the Université de Moncton was consulted by the first author to guide the development of the literature search strategy. The search was structured using the PICO framework, focusing on undergraduate nursing students (Population), transition from university-based simulation learning (Intervention), with or without comparison groups (Comparison), and outcomes related to well-being, adaptability, and sense of belonging (Outcomes). Based on prior knowledge and a preliminary search, a list of relevant keywords and synonyms was developed for each concept. This list was expanded using Medical Subject Headings (MeSH) terms and proximity operators where applicable. The finalized search strategy (Multimedia Appendix 1) was first tested in CINAHL to ensure it captured relevant studies, then adapted for other databases including PubMed, APA PsycINFO, Scopus, and ScienceDirect. Reference lists of included studies will be reviewed, and additional articles may be identified through snowball sampling during the data extraction phase.

### Data Management

Following the literature search conducted in collaboration with the academic librarian, all identified references were first imported into the bibliographic management software Zotero [18]. From there, the records were exported and uploaded into Covidence [19] for screening. Duplicate entries were automatically removed by the Covidence system prior to the title and abstract screening process.

### Study Selection

All studies will be reviewed to determine whether they meet the inclusion criteria defined in the Covidence software. The screening process will begin with a review of titles and abstracts, as outlined in the standard scoping review methodology. If the abstract is not immediately available but the title appears potentially relevant, the study will be advanced to the next stage for a full-text review. Titles and abstracts will be screened independently by two reviewers, blinded to each other’s decisions. Studies will proceed to full-text screening when both reviewers agree, and any conflicts will be resolved by a third reviewer. Full-text screening will also be conducted independently by two blinded reviewers using Covidence, with disagreements resolved by a third blinded reviewer. Studies will be excluded at this stage if they do not meet all inclusion criteria, and reasons for exclusion will be documented and reported in the final review. The study selection process and results will be fully described in the final scoping review and illustrated using a PRISMA-ScR flow diagram. Additionally, a list of excluded studies along with the reasons for exclusion will be provided.

### Data Extraction

Data will be extracted from the included studies using a structured tool developed by the review team. The preliminary version of the data extraction tool (Multimedia Appendix 2) will be pilot-tested on two studies by all reviewers before full implementation.

To address conceptual overlap across studies, operational definitions for well-being, adaptability, and sense of belonging will be established a priori and used to guide data extraction. Given the documented overlap among well-being, resilience, adaptability, and coping-related constructs [20], particular attention will be paid to authors’ conceptual definitions, theoretical frameworks, and measurement instruments. Constructs such as resilience or flexibility will be coded separately and mapped analytically to the predefined constructs only when explicit conceptual alignment is supported by the authors’ definitions or measurement tools. During data extraction, findings will be categorized according to the construct explicitly targeted by each study, rather than interchangeable terminology.

To ensure reliability, two reviewers will independently extract data from each study. A third member of the research team will verify the extracted data and support discrepancy resolution through discussion. The extraction tool may be refined iteratively, with any modifications documented in the final review.

### Data Analysis

Extracted data will be analyzed using a combination of descriptive statistics and narrative synthesis. Descriptive analyses will summarize the number of included studies, publication years, countries of origin, and target populations, which will be presented in tabular format.

The narrative synthesis will address the review objectives and summarize findings related to university-based simulation learning, transition support, well-being, adaptability, and sense of belonging, drawing on both qualitative and quantitative evidence. Overlapping constructs will be examined comparatively and acknowledged explicitly to preserve conceptual nuance, maintain analytical coherence, and accurately reflect the conceptual complexity of the field.

Thematic coding will be applied to categorize types of simulations, educational interventions, and outcomes to support synthesis and interpretation. Barriers and facilitators influencing outcomes will be identified, intervention effectiveness will be examined where applicable, and gaps in the literature will be highlighted. Themes will be developed through an iterative, inductive coding process and organized into higher-order categories through cross-study comparison. Coding will be reviewed collaboratively by the research team to ensure analytic rigor and consistency.

Credibility will be strengthened through duplicate coding and consensus-based discussions. Dependability and confirmability will be supported by a transparent, iterative analytic process with documented analytic decisions, while transferability will be facilitated through detailed reporting of study contexts and characteristics. Discrepancies will be resolved through consensus or consultation with a third reviewer when necessary.

Consistent with scoping review methodology, the methodological quality of included studies will not be formally assessed, as the purpose of this review is to map the breadth of available evidence.

### Ethical Considerations

In accordance with institutional and national guidelines, research ethics approval was not sought for this study because it consists of a scoping review of publicly available literature. The study did not involve human participants, identifiable personal information, or primary data collection.

## Results

This study was funded by the Faculty of Graduate Studies and Research at the Université de Moncton, with funding received in May 2025. The study was initiated in January 2025, and the final version of the protocol was completed in June 2025. A preliminary search of the published literature (excluding gray literature) was conducted in September 2025, resulting in 1194 articles after duplicate removal.

The formal literature search began in October 2025, with results expected by June 2026. We plan to submit a manuscript to *JMIR Nursing* to share our findings with an academic audience, aiming for submission in the summer of 2026. Additionally, we intend to present the results at conferences to promote broader dissemination. These preliminary results are provisional and will be updated following completion of the final search strategy.

## Discussion

### Expected Insights

This scoping review is anticipated to provide a structured synthesis of how undergraduate nursing students’ psychosocial experiences are conceptualized, measured, and supported during the transition from university-based simulation learning to clinical practice. Preliminary examination of the literature suggests that, although simulation-based learning is well established as a strategy for developing technical competence and clinical reasoning, psychosocial dimensions of transition—in particular, well-being, adaptability, and sense of belonging—remain inconsistently defined and variably operationalized. By systematically mapping definitions, measurement tools, and interventions, the review will clarify how constructs such as well-being, adaptability, and sense of belonging are conceptualized and operationalized across diverse studies. Consistent with prior work, preliminary examination indicates that psychosocial readiness receives less explicit attention than technical competence, often appearing as a secondary or implicit outcome. This gap highlights the need for a structured overview to inform educational strategies and support frameworks.

### Implications for Nursing Education

The findings will have direct implications for curriculum design and pedagogical approaches. Identifying interventions, such as structured debriefing, mentorship programs, and orientation sessions, that effectively promote resilience, emotional stability, and social integration will enable educators to implement evidence-based practices. In addition, mapping the range of measurement approaches, including self-report instruments, reflective tools, and emerging digital assessment methods, will clarify how psychosocial outcomes are evaluated and how these tools align with educational objectives and simulation-based pedagogy. Strengthening these dimensions is essential not only for academic success but also for patient safety and professional identity formation. By synthesizing diverse strategies and outcomes, this review will contribute to the development of transition models that integrate both technical and psychosocial aspects of clinical learning.

### Research Gaps and Future Directions

The review is expected to reveal significant gaps, including inconsistent definitions of key constructs, variability in measurement approaches, and limited evaluation of intervention effectiveness. Future research should prioritize longitudinal studies to assess the sustained impact of transition support strategies and explore innovative approaches, such as peer-assisted learning or digital tools, to enhance adaptability and belonging. Additionally, cultural and contextual factors influencing students’ experiences should be examined to ensure interventions are applicable across different educational settings.

This protocol addresses a critical yet underexplored dimension of nursing education by systematically mapping the psychosocial factors of well-being, adaptability, and a sense of belonging during the transition from university-based simulation learning to clinical practice. The absence of existing reviews on this topic underscores its originality and the need for evidence-informed strategies to support students in navigating one of the most challenging phases of their academic journey.

## Supplementary material

10.2196/86813 Multimedia Appendix 1 Search strategy.

10.2196/86813 Multimedia Appendix 2 Data extraction tool.

10.2196/86813 Checklist 1 PRISMA-ScR checklist.

## Abbreviations

MeSH: Medical Subject Headings
PRISMA-ScR: Preferred Reporting Items for Systematic Reviews and Meta-Analyses extension for Scoping Reviews
