# Supplementary material for: Exploring the Well-Being, Adaptability, and Sense of Belonging of Undergraduate Nursing Students During the Transition From Simulation to Clinical Practice: Protocol for a Scoping Review
Source: JMIR Res Protoc. 2026 Feb 13;15:e86813. doi: 10.2196/86813 (PMC12904347; doi:10.2196/86813)
Supplement: Multimedia Appendix 1 [file resprot-v15-e86813-s001.docx]

***CINAHL***

(XB laborator* OR MH "Clinical Laboratories" OR MH "Laboratories" OR MH "Simulations+" OR XB simulat*) AND (MH "Learning Environment, Clinical" OR MH "Clinical Competence+" OR MH "Education, Clinical+" OR MH "Student Placement" OR MH "Career Planning and Development" OR MH "Outcomes of Education" OR XB "Clinical Learning" OR XB "Clinical education" OR XB "Clinical competenc*" OR MH "Internship and Residency+" OR XB internship OR XB "student placement") AND (XB transition OR MH "Psychological Well-Being" OR XB "well being" OR MH "Wellness Evaluation" OR MH "Adaptation, Psychological+" OR XB adaptation OR XB adapting OR XB belonging OR XB “student experience” OR MH "Student Experiences" OR MH "Experiential Learning" OR XB “experiential learning” OR MH “Hardiness” OR XB Resilien* OR XB wellness) AND ((*XB Nurs* AND XB student*) OR MH "Students, Nursing+" OR MH "Education, Nursing+")

***PubMed***

("Laboratories"[Mesh] OR "Laboratories, Clinical"[Mesh] OR laborator*[Title/Abstract] OR simulat*[Title/Abstract] OR "Simulation Training"[Mesh]) AND ("Clinical Competence"[Mesh] OR "Clinical Clerkship"[Mesh] OR "clinical education"[Title/Abstract] OR "clinical learning"[Title/Abstract] OR "clinical competenc*"[Title/Abstract] OR "Internship and Residency"[Mesh] OR internship[Title/Abstract] OR "student placement"[Title/Abstract]) AND ("Psychological Well-Being"[Mesh] OR transition[Title/Abstract] OR well being[Title/Abstract] OR wellness[Title/Abstract] OR "Adaptation, Psychological"[Mesh] OR adaptation[Title/Abstract] OR adapting[Title/Abstract] OR belonging[Title/Abstract] OR "student experience"[Title/Abstract] OR "experiential learning"[Title/Abstract] OR "Resilience, Psychological"[Mesh] OR resilien*[Title/Abstract]) AND ("Students, Nursing"[Mesh] OR "Education, Nursing"[Mesh] OR (nurs*[Title/Abstract] AND student*[Title/Abstract]))

***Scopus***

((TITLE-ABS-KEY ( transition )) OR (TITLE-ABS-KEY ("well being")) OR (TITLE-ABS-KEY (adaptation)) OR (TITLE-ABS-KEY (adapting)) OR (TITLE-ABS-KEY (belonging)) OR (TITLE-ABS-KEY (student W/3 experience)) OR (TITLE-ABS-KEY ("experiential learning")) OR (TITLE-ABS-KEY (resilien*)) OR (TITLE-ABS-KEY (wellness)) OR (INDEXTERMS ("well being")) OR (INDEXTERMS ("wellness")) OR (INDEXTERMS ("adaptation")) OR (INDEXTERMS ("Student experiences")) OR (INDEXTERMS ("Experiential learning")) OR (INDEXTERMS ("Hardiness"))) AND ((INDEXTERMS (Clinical learning environment)) OR (INDEXTERMS (Clinical competence)) OR (INDEXTERMS (Clinical education)) OR (INDEXTERMS (Student placement)) OR (INDEXTERMS (Career planning)) OR (INDEXTERMS (Outcomes of education)) OR (INDEXTERMS (internship)) OR (TITLE-ABS-KEY (Clinical W/3 learning)) OR (TITLE-ABS-KEY (Clinical W/3 education)) OR (TITLE-ABS-KEY (Clinical W/3 competenc*)) OR (TITLE-ABS-KEY (internship)) OR (TITLE-ABS-KEY ("student placement"))) AND ((INDEXTERMS ("laboratory" OR "laboratories")) OR (TITLE-ABS-KEY (simulat*)) OR (INDEXTERMS ("simulation")) OR (TITLE-ABS-KEY (laborator*))) AND ((TITLE-ABS-KEY (nurs* AND student*)) OR (INDEXTERMS ("Nursing Students")) OR (INDEXTERMS ("Nursing education"))) AND PUBYEAR > 2014 AND PUBYEAR < 2027 AND (LIMIT-TO (DOCTYPE , "ar") OR LIMIT-TO (DOCTYPE , "re")) AND (LIMIT-TO ( LANGUAGE , "French") OR LIMIT-TO (LANGUAGE , "English"))

***APA PsycINFO***

(XB laborator* OR DE "Educational Laboratories" OR DE "Simulation" OR XB simulat*) AND (DE "Career Development" OR XB "Clinical Learning" OR XB "Clinical education" OR XB "Clinical competenc*" OR XB internship OR XB "student placement") AND (XB transition OR DE "Well Being" OR XB "well being" OR DE "Adaptation" OR XB adaptation OR XB adapting OR XB belonging OR XB “student experience” OR DE "Experiential Learning" OR XB “experiential learning” OR DE "Resilience (Psychological)" OR XB Resilien* OR XB wellness) AND ((*XB Nurs* AND XB student*) OR DE "Nursing Students" OR DE "Nursing Education")

***Science Direct***

(laboratory OR simulation) AND (clinical) AND (wellness OR belonging OR adaptation OR transition) AND ("nursing student" OR "nurse education") Advanced search - Show all fields - Title, abstract or author-specified keywords
