# Supplementary material for: Exploring the Well-Being, Adaptability, and Sense of Belonging of Undergraduate Nursing Students During the Transition From Simulation to Clinical Practice: Protocol for a Scoping Review
Source: JMIR Res Protoc. 2026 Feb 13;15:e86813. doi: 10.2196/86813 (PMC12904347; doi:10.2196/86813)
Supplement: Multimedia Appendix 2 [file resprot-v15-e86813-s002.docx]

**Data Extraction Elements**

| **Study ID** | Author(s) and year of publication |
| --- | --- |
| **Full Citation** | Complete reference (APA or other style) |
| **Country / Setting** | Country and type of institution (University, hospital, simulation lab) |
| **Study Design** | Quantitative, qualitative, mixed-method, review |
| **Population** | Type of students (undergraduate), sample size, year of study |
| **Simulation Intervention** | Type of simulation, duration, frequency |
| **Transition Support / Intervention** | Orientation, mentoring, debriefing, other pedagogical interventions |
| **Comparison / Control** | If applicable: students without simulation or with alternative transition support |
| **Outcomes Measured** | Well-being (stress, anxiety, burnout), adaptability / coping / resilience, sense of belonging / social integration / inclusion |
| **Measurement Tools** | Scales, questionnaires, interviews, observations, others |
| **Key Findings – Well-being** | Summary of findings related to well-being (qualitative/quantitative) |
| **Key Findings – Adaptability** | Summary of findings related to adaptability (qualitative/quantitative) |
| **Key Findings – Belonging** | Summary of findings related to sense of belonging (qualitative/quantitative) |
| **Barriers / Facilitators** | Factors identified that hinder or promote well-being, adaptability, or belonging |
| **Intervention Effectiveness** | If applicable, impact or effectiveness of educational intervention |
| **Gaps / Recommendations** | Literature gaps and authors’ recommendations for future research |
| **Notes / Comments** | Additional observations relevant for synthesis |
